# L‐DOPA Test in the Diagnosis of Childhood Short Stature: Evaluation of Growth Hormone Peaks Over Time

**DOI:** 10.1002/edm2.70000

**Published:** 2024-08-30

**Authors:** Barbara Castelli, Rita De Santis, Simona Carrera, Marco Andrea Malanima, Salvatore De Masi, Stefano Stagi

**Affiliations:** ^1^ Health Sciences Department University of Florence Florence Italy; ^2^ Meyer Children's Hospital IRCCS Florence Italy; ^3^ Clinical Trial Center Careggi University Hospital Florence Italy

**Keywords:** children, growth hormone, L‐DOPA

## Abstract

**Introduction:**

In childhood, growth hormone (GH) deficiency (GHD) diagnosis is based on auxological assessment and biochemical provocative tests, whose reliability remains disputed. Recently, several papers have been published on standardising the duration of some tests. The aim of our study was to analyse the possible length reduction of the L‐DOPA provocative test.

**Methods:**

We retrospectively investigated the response of GH to L‐DOPA in 256 children, analysing 267 tests (some patients were retested over time for the persistence of severe auxopathy). We studied the same data considering GH peak threshold both at 8 ng/mL (Italian GHD cut‐off) and at 10 ng/mL (international cut‐off). Based on stimulation tests, patients were divided into two groups: GHD and no‐GHD short children. We described the results in the whole population and then clustering for gender and pubertal stage. We termed as index the test stopped at 90 min.

**Results:**

The GH peak after L‐DOPA mostly occurred at 60 min. The sensitivity of the index test was the highest, while the specificity was slightly higher using the 8 ng/mL threshold (specificity = 0.68; 95% CI 0.60–0.76) then using the 10 ng/mL threshold (specificity = 0.56; 95% CI 0.47–0.65) at 90 min. The two ROC curves showed moderate performance of the test at 90 min. While the negative predictive value was 100% in both tests, the positive predictive value was slightly better with 10 ng/mL cut‐off. Considering the two groups established by GHD definition and placing a GH threshold at 10 ng/mL, stopping L‐DOPA test time at 90 min would have changed the test result and subsequentially patient's classification in 3/267 of the analysed tests (1.1%), while with the Italian GH threshold value at 8 ng/mL in 7/267 of the tests (2.6%).

**Conclusions:**

Our research shows that omitting 120‐min time reduces L‐DOPA test specificity, especially with GHD cut‐off at 10 ng/mL.

## Graphical abstract

In childhood growth hormone deficiency (GHD) diagnosis is based on auxological assessment and biochemical provocative tests. We analysed the L‐DOPA provocative test, evaluating GH peaks distribution over time and examining in detail the 120‐min time, to assess the possibility of length reduction. We noticed that omitting 120‐min time reduces test specificity, especially with GHD cut‐off at 10 ng/mL.

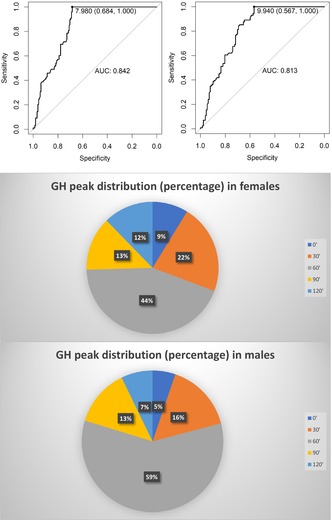

## 1 Introduction

Growth hormone (GH) deficiency (GHD) diagnosis in childhood is based on clinical and auxological assessments, combined with biochemical tests [1]. GHD may present as an isolated problem or in combination with multiple pituitary hormone deficiencies [1]. Currently, although most studies show poor sensitivity and specificity, in children GH stimulation tests are fundamental in diagnosing GHD because of the pulsatile nature of GH production, which means its random measurement cannot be used for diagnostic purposes [2, 3]. A 12‐ or 24‐h GH profile may have a role in the diagnosis of GHD by reducing false‐positive stimulation tests [2, 4]. In provocative tests a stimulus for GH production is administered parenterally or orally and the GH response is subsequently evaluated. The protocols for GHD diagnosis vary substantially in European countries and in the United States [5]. No single test is sensitive and specific enough to confirm a diagnosis [5]. Conventional GH stimulation tests have low specificity and poor reproducibility [5, 6] and results can be influenced by factors such as age, gender, puberty, nutritional status and body weight, which modulate the secretion of GH [7]. Clinicians can improve the reliability of testing by taking into account these factors [8]. Moreover stimulation tests may have side effects because of the drugs used as stimuli [3]. Different pharmacological agents generate different mean GH peak sizes. In suspected isolated GHD, two different GH stimulation tests are required and both tests must be abnormal to define the presence of a GH deficiency [1, 9]. There is no consensus on a specific GH cut‐off value and priming with sex steroids remains a matter of debate for the subsequent interpretation of the test outcome [1, 5]: GH peaks are considerably higher in puberty due to increases in sex hormones [2]. The Growth Hormone Research Society recommends the following GH stimulation tests: insulin, clonidine, arginine, glucagon, L‐DOPA [1]. In particular, L‐DOPA stimulates the dopaminergic and adrenergic secretion of GH [10]. Side effects are nausea, vomiting and dizziness. GH‐releasing hormone (GHRH) provocative test can potentially differentiate between patients with pituitary dysfunction and patients with hypothalamus abnormalities [11]. L‐DOPA, the metabolic precursor of dopamine (DA), has primarily been used for the treatment of Parkinson's disease (PD), crossing the blood–brain barrier and replacing the DA that would be normally released by the substantia nigra DA pathway [12]. Data on the pharmacokinetics of the oral L‐DOPA formulation are reported on PD studies [13].

Currently, a child with clinical criteria for GHD and a peak GH concentration of less than 10 ng/mL in two different tests is diagnosed with GHD [1]. Recently it has been suggested that the threshold should be lowered to 7 ng/mL, depending on the assay [14]. In 2014, cut‐off values were changed in Italy. AIFA (Agenzia Italiana del farmaco) in Note 39 considers children who have a GH response <8 ng/mL in two different pharmacological tests performed on different days as GH deficient [15]. A number of works have been published on standardising the duration of the test for some stimuli which would reduce healthcare costs [16].

Up to now, during an L‐DOPA stimulation test, the dosage of GH has been performed after 30, 60, 90 and 120 min [17]. A few data exist on the analysis of this test. Therefore, the aim of our study was to evaluate the distribution of GH peaks during L‐DOPA test examining results at 90 min compared with the reference test at 120‐min time.

## 2 Materials and Methods

We retrospectively and observationally investigated the response of GH to the L‐DOPA stimulus in 256 children (147 males, 109 females, 177 pre‐pubertal and 79 pubertal), referred for short stature to the Auxo‐endocrinological Unit at Meyer Children's University Hospital IRCCS in Florence (Italy) between January 2018 and January 2021.

The inclusion criteria were height less than −2 SD below the mean for the normal population and / or a subnormal growth velocity [18]. Exclusion criteria were genetic, oncological or neurological diseases. Pubertal status was assigned using Tanner staging [19, 20]. Height, height velocity and weight were evaluated according to international growth references [18]. Bone age was estimated from an x‐ray of the left wrist and hand, using the atlas technique of Greulich and Pyle [21].

L‐DOPA was orally administered at 10 mg/kg and blood samples were measured at baseline (T0), 30 (T30), 60 (T60) and 120 (T120) minutes.

IGF‐I, IGFBP‐3 and GH levels were determined using a chemiluminescent enzyme immunoassay with the Immulite Analyzer (Immulite 2000, Diagnostic Product Corporation, Los Angeles, Calif., United States). The intra‐ and inter‐assay variation coefficients regarding IGF‐I, IGFBP‐3 and GH were 2.3–3.9, 3.7–8.1; 4.6–6, 6.8–9.5 and 3.80% and 6.40%, respectively.

Based on the results of the stimulation tests, patients were divided into two groups. The first group consisted of patients with a GHD diagnosis based on height and/or growth rate 2 SD below average for normal population and a GH peak <8 ng/mL after two provocative tests with different stimuli on two separate occasions. The second group included patients with a diagnosis of non‐GHD (no‐GHD) short stature based on the same auxological criteria, but with at least one normal response to provocative tests or who had undergone only a first provocative test with L‐DOPA stimulus during the considered period (25 patients). We analysed the same data considering GH peak threshold value at 10 ng/mL (international cut‐off). We termed as peak the highest reached GH value.

We used the threshold of 8 ng/mL as the index test when evaluated at 90 min and compared it with the reference standard defined as a threshold value of 8 ng/mL at 120 min observed in both L‐DOPA and arginine administration test. Then, we calculated the sensitivity, specificity, and predictive value of the index test. We applied the same method with a threshold of 10 ng/mL (index test at 90 min vs. reference standard at 120 min).

Finally, we generated the ROC curves by testing the diagnostic significance of the different thresholds at 90 min compared to the reference standard with a threshold value of 8 ng/mL and 10 ng/mL at 120 min.

The study protocol was approved by the A. Meyer Children's Hospital Ethics Committee, and informed consent was obtained from the children's parents (research project number 82/2021).

## 3 Results

Table 1 describes baseline characteristics of patients in our study.

**TABLE 1:** Baseline characteristics of patients in our study considering GHD threshold 8 ng/mL.

	GHD (98)	No‐GHD (158)	*p*
Age, years	9.57 ± 3.22	9.88 ± 3.13	NS
Sex, M/F	55/43	92/66	NS
Height, SDS	−2.01 ± 0.65	−2.28 ± 0.74	<0.005
BMI, SDS	17.18 ± 3.32	15.97 ± 2.48	<0.005
Bone age, years	7.38 ± 3.40	7.74 ± 3.10	NS
GH peak, ng/mL	4.68 ± 2.04	10.24 ± 4.87	<0.0001

Our data indicate that 90.6% of the GH peaks after L‐DOPA, in both GHD and no‐GHD patients, occurred before 120 min, usually at 60 min.

In our study, L‐DOPA test was most frequently performed as a second test after an arginine test. The mean time between the two tests performance was 128 days.

The diagnostic accuracy of the index tests (based on the peak value of 8 and 10 ng/mL after 90 min) for a total of 256 tested patients is shown in Table 2. The sensitivity was by definition the highest, while the specificity was slightly higher using the threshold value of 8 ng/mL (specificity = 0.68; 95% CI 0.60–0.76) then using the threshold value of 10 ng/mL (specificity = 0.56; 95% CI 0.47–0.65) at 90 min.

**TABLE 2:** Diagnostic measures of GH test at 90 min with cutoffs 8 ng/mL and 10 ng/mL, respectively.

	Patients with GH deficit	Patients without GH deficit	
GH test <8 at 90 min	98	50	148
GH test ≥8 at 90 min	0	108	108
Total	98	158	256

		*p*‐Values*
Sensitivity, 95% CI	98/98 = 1.0 (0.97,1.0)	1
Specificity, 95% CI	108/158 = 0.68 (0.60, 0.76)	<0.001
Predictive positive value, 95% CI	98/148 = 0.66 (0.58, 0.74)	<0.001
Predictive negative value, 95% CI	108/108 = 1.00 (0.97, 1.00)	1

	Patients with GH deficit	Patients without GH deficit	
GH test <10 at 90 min	129	56	185
GH ≥10 at 90 min	0	71	71
Total	129	127	256

		*p*‐Values*
Sensitivity, 95% CI	129/129 = 1.0 (0.97, 1.0)	1
Specificity, 95% CI	71/127 = 0.56 (0.47, 0.65)	<0.001
Predictive positive value, 95% CI	129/185 = 0.70 (0.63, 0.76)	<0.001
Predictive negative value, 95% CI	71/71 = 1.00 (0.95, 1.00)	1

* *p*‐Values are referred to the comparisons between index test and reference test accuracy measures.

**FIGURE 1:**
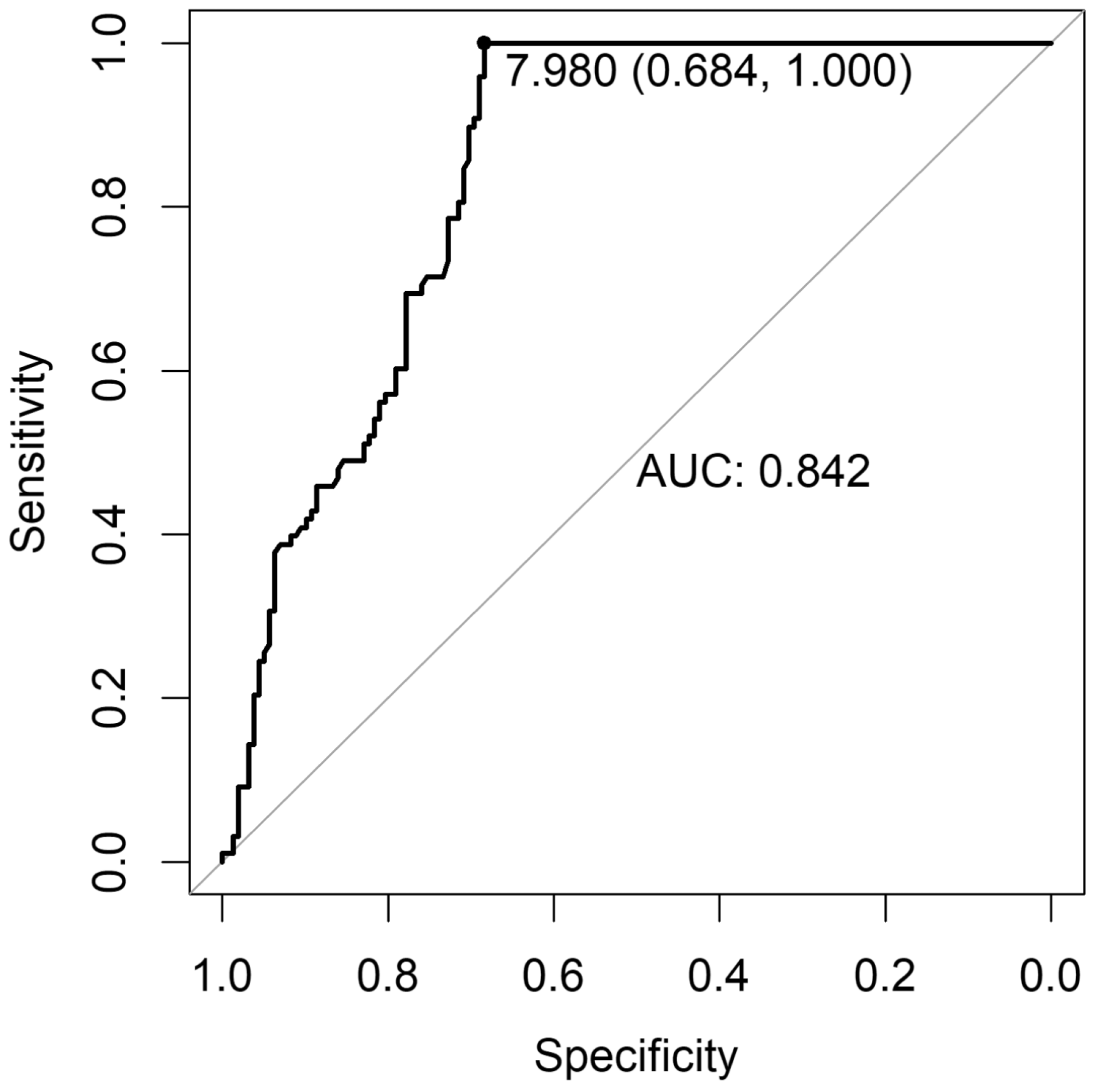
ROC curve of index test at 90 min (GH threshold at 8 ng/mL). The point corresponding to the maximum Youden index is illustrated.

**FIGURE 2:**
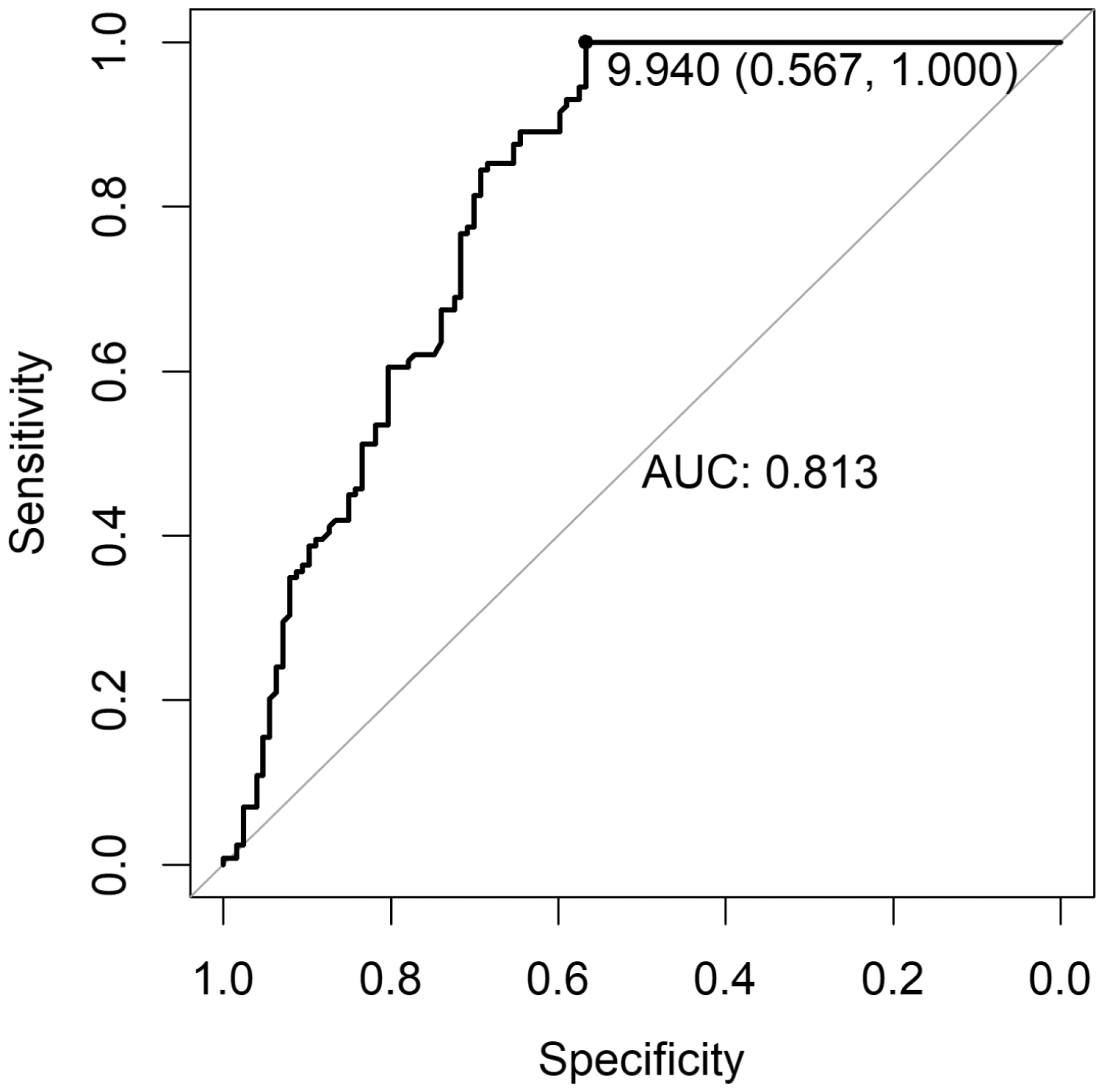
ROC curve of index test at 90 min (GH threshold at 10 ng/mL). The point corresponding to the maximum Youden index is illustrated.

The two ROC curves (Figures 1 and 2), based on the reference values defined as a threshold of 8 ng/mL (Figure 1) and of 10 ng/mL (Figure 2) evaluated at 120 min, both showed moderate performance of the test at 90 min (AUC = 0.842; 95% CI 0.795–0.889, reference <8 ng/mL; AUC = 0.813; 95% CI 0.756–0.867, reference <10 ng/mL).

While the negative predictive value (NPV) was 100% in both tests, the positive predictive value (PPV) was slightly better when using the cut‐off value of 10 ng/mL (Table 2), even if the specificity was lower.

Using a GH threshold for the diagnosis of GHD at 8 ng/mL (the Italian cut‐off), including all the patients who performed the L‐DOPA test in the referred period due to suggestive characteristics and considering the standard definition of GHD with two different deficient GH stimulation tests, 98 patients received a final diagnosis of GHD (55 males and 43 females, mean age 9.57 ± 3.22 years) and 158 patients received a final diagnosis of no‐GHD short stature (92 males and 66 females, mean age 9.88 ± 3.13 years). 178/256 were pre‐pubertal and 78/256 pubertal, without significant difference between GHD and no‐GHD in the ratio pre‐pubertal/pubertal (Table 1). Furthermore, 11/158 no‐GHD short children underwent over time more than one L‐DOPA test for persistence of their severe auxopathy; therefore, in the no‐GHD short children cohort a total of 169 L‐DOPA tests were performed.

In the GHD group, 11/98 of the tests (11.22%) had the highest GH value at 120 min, although obviously lower than 8 ng/mL.

In the no‐GHD cohort (169 tests), 14/169 of the tests (8.28%) had the peak value (the highest value) at 120 min. Analysing these 14 tests, 7/14 reached at 120 min the first threshold and peak value, whereas 2/14 presented a first threshold value before 120 min with the peak value at 120 min. The peak value at 120 min was below the cut‐off for 5/14 of the analysed group, but these patients were diagnosed as no‐GHD in a following non deficient test with a different stimulus. The high frequency of false‐positives in GH stimulation tests is the main reason in literature for performing two stimulation tests, avoiding overdiagnosis of GHD [22]. Considering the two groups in the whole, 7/267 of the performed tests (2.62%, all in the no‐GHD short children group) showed a GH peak at 120 min, potentially changing the result of the test.

Bringing the GHD threshold at 10 ng/mL, 129 patients would have fallen into the GHD group (133 L‐DOPA tests), whereas 127 would have been diagnosed as no‐GHD short children (134 L‐DOPA tests). In this case, in the GHD group 14/133 of the tests (10.5%) had a GH peak at 120 min and in the no‐GHD short children 11/134 of the tests (8.2%). Among these last‐mentioned tests, 3/11 presented at 120 min the first threshold value coinciding with the peak value, whereas 1/11 with 120 GH peak presented the first threshold value before 120 min and 7/11 patients did not reach the cut‐off at this test but were diagnosed as no‐GHD in a subsequent non deficient test with a different stimulus. So, considering the performed tests in the whole, 3/267 (1.1%, all in the no‐GHD short children group) had a GH peak at 120 min, potentially changing the test results.

**FIGURE 3:**
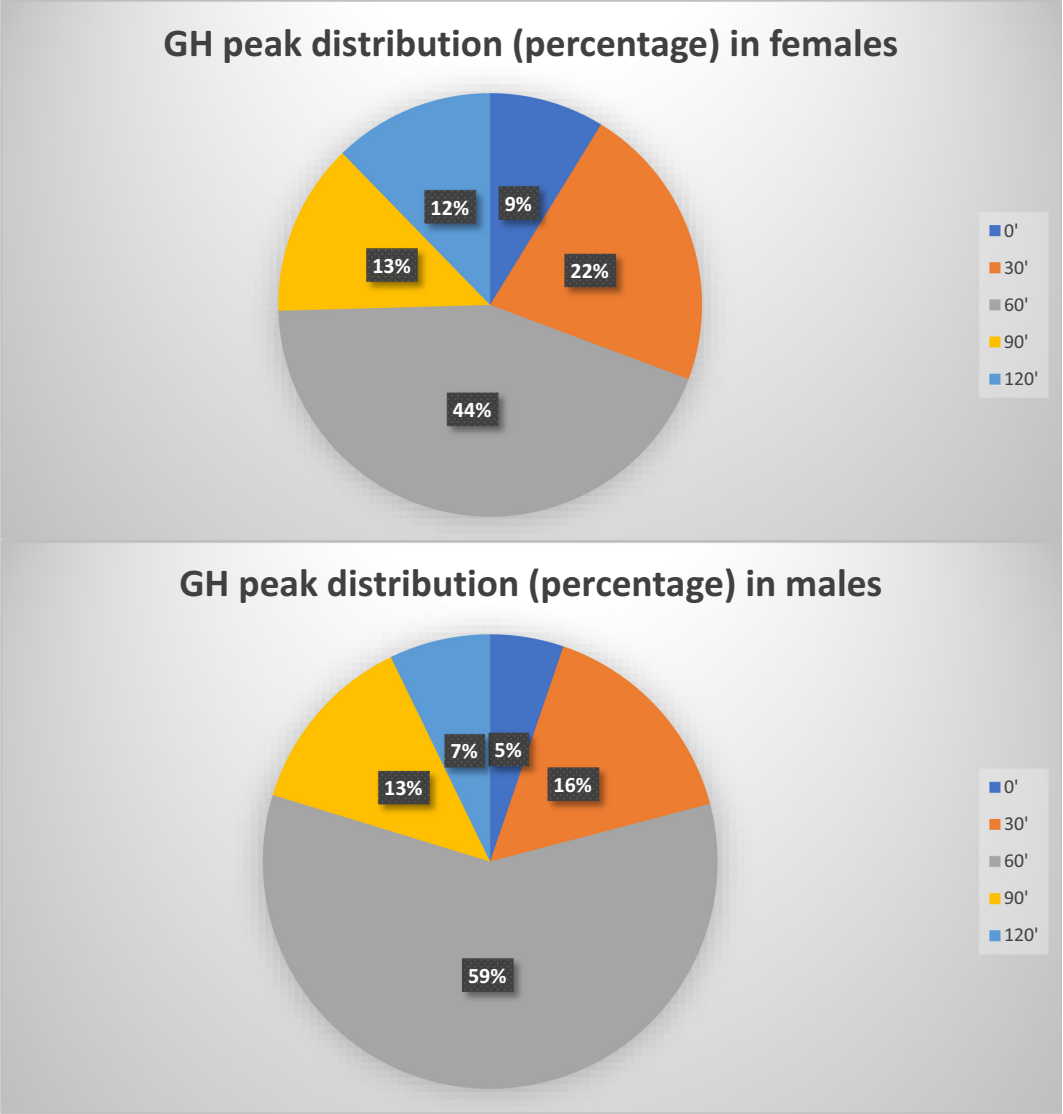
GH peak distribution in females and males.

At the GH threshold for GHD diagnosis at 8 ng/mL, 86 were pre‐pubertal females (35 GHD and 51 no‐GHD short children), 23 pubertal females (8 GHD and 15 no‐GHD short children), 91 pre‐pubertal males (36 GHD and 55 no‐GHD short children) and finally 56 were pubertal males (19 GHD and 37 no‐GHD). There was a significant difference in the percentage of tests with GH peak at 120 min between pre‐pubertal GHD females (11.5%) and males (5.5%; *p* < 0.05) and no‐GHD short children pre‐pubertal females (12.5%) and males (7.0%; *p* < 0.05). This difference was also evident between pubertal GHD females (25%) and males (15.8%; *p* < 0.05), but not no‐GHD short children pubertal females (6.7%) and males (4.9%). Table 3 and Figure 3 describe the distribution of peaks clustering the two groups for gender and pubertal status.

**TABLE 3:** GH peaks distribution in 267 tests.

Time (min)	Pre‐pubertal		Pubertal	
	GHD *n* tests (%)	No‐GHD *n* tests (%)	GHD *n* tests (%)	No‐GHD *n* tests (%)
Females				
0	4 (11)	5 (9)	0 (0)	1 (7)
30	9 (26)	13 (23)	2 (25)	1 (7)
60	15 (43)	26 (46)	3 (38)	6 (40)
90	3 (9)	5 (9)	1 (12)	6 (40)
120	4 (11)	7 (13)	2 (25)	1 (7)
Males				
0	2 (6)	3 (5)	3 (16)	0 (0)
30	4 (11)	10 (18)	3 (16)	7 (17)
60	21 (58)	35 (61)	9 (47)	25 (61)
90	7 (19)	5 (9)	1 (5)	7 (17)
120	2 (6)	4 (7)	3 (16)	2 (5)

## 4 Discussion

Our evaluation on GH peaks distribution in 267 tests (256 children) suggests that on some occasions the GH response at 120‐min changes test result. Therefore, although most of the time the 120‐min sample has little impact, it is not possible to omit it.

Testing for GHD after L‐DOPA stimulus is widely used in the diagnostic process of short stature [23]. Currently, even if the used provocative agents have low specificity and poor reproducibility [5, 6], stimulation tests are essential for GHD diagnosis [7]. Even though the agreed GHD cut‐off is 10 ng/mL [7, 24], the ideal threshold for pharmacological stimuli which distinguishes between GH deficient and sufficient children has not been established [7]. There are several limitations on results interpretation and lack of standardisation [25].

Whereas literature is still scarce, we performed an analysis of GH peaks over time after L‐DOPA stimulus. This test has the advantage of the oral administration of the drug.

In our cohort, in both GHD and no‐GHD patients the GH peak after L‐DOPA stimulus mostly occurred before 120 min, usually at 60 min. It was reported that the 60 min sample was also the best single sample to rule out GHD after clonidine stimulation [26].

In the no‐GHD short children group, at 8 ng/mL GHD cut‐off, 2.6% of tests (7/267) really showed a GH peak at 120 min and at 10 ng/mL GHD cut‐off 1.1% of tests (3/267). These cases would have had result modification by time test reduction, and subsequently their classification would have been affected.

We calculated the sensitivity and specificity of the L‐DOPA test at 90 min using the peak value obtained at 120 min by L‐DOPA and arginine test as the reference standard. The specificity of the two cut‐off values used was rather low, especially when we used the value of 10 ng/mL as the threshold below which the test is considered positive. In any case, the positive predictive value was higher for the test with a threshold value of 10 ng/mL at 90 min, which is ascribed to the higher prevalence of disease achieved by the reference test with a threshold value of 10 ng/mL at 120 min. In last years, great interest emerged in shortening provocative test duration. Various stimulus tests have been re‐evaluated to find the best timing for the diagnosis of GHD, without any misdiagnoses of GHD or no‐GHD short children, helping to save healthcare costs and reduce the patient's and their family's time in hospital [16]. The arginine test, the clonidine test and the glucagon test have been re‐evaluated and a reduction of test time proposed [10, 16, 27]. We reported that the arginine test can be administered for only 90 min without significantly changing its validity [10]. Similarly, for glucagon, Strich, Terespolsky and Gillis [27] proposed a reduction in measurements of GH to 150 min. They analysed 222 tests and only three showed a GH peak at 180 min [27]. In 2013, Christoforidis et al. [28] retrospectively evaluated the possibility to reduce the number of GH analyses during clonidine and glucagon tests without compromising accuracy. In 2015, Al Khalifah, Moisan and Bui [29] examined the specificity of both tests using a shorter duration of timed samples: 90 min for clonidine and 60 min for arginine. In 2016, Gillis et al. [30] confirmed that clonidine stimulation tests is equally effective when terminated at 90 min from stimulation. Recently, Fatani [31] concluded that 0‐min time point could be eliminated without compromising the combined GH stimulation tests diagnostic value, thus resulting in cost reduction. Jaruratanasirikul, Leethanaporn and Sriplung [32] proposed shortening the insulin test to 120‐min sampling. Reducing test length can cut costs and time spent in hospital [32], which is particularly important in resource‐limited settings [26].

It was postulated that the presence of atypical peak GH timing may be a factor that predicts lower growth hormone velocity during the first year of recombinant GH treatment in pre‐pubertal children with GHD [33].

Interestingly, in our study, for GHD and no‐GHD females, there were significantly different percentages of tests with GH peak at 120 min. This may be explained by gender differences in L‐DOPA pharmacokinetics, as reported in L‐DOPA‐naïve patients with Parkinson's disease [34]. The significance of this aspect on the sensitivity of the test and on the specificity of a diagnosis of GHD has yet to be studied.

Our data showed that most patients had the GH peak before 120 min, usually at 60 min.

In our research study, as previously assessed for other provocative tests, we reflected on the possibility to shorten the duration of the L‐DOPA provocative test. We found that omitting 120‐min time reduces test specificity, especially with GHD cut‐off at 10 ng/mL, changing test result. Therefore, from our data, it is not possible to reduce the length of time of the L‐DOPA test. However, this was a single centre data collection. Further studies are expected on GH response to stimuli over time, to better standardise provocative tests for the diagnosis of short stature in children.

## Author Contributions

**Barbara Castelli:** conceptualization (lead), data curation (lead), formal analysis (lead), methodology (lead), resources (lead), writing – original draft (lead). **Rita De Santis:** conceptualization (equal), validation (equal), writing – review and editing (equal). **Simona Carrera:** data curation (equal), writing – review and editing (equal). **Marco Andrea Malanima:** data curation (supporting), formal analysis (supporting), methodology (supporting), writing – review and editing (equal). **Salvatore De Masi:** data curation (supporting), formal analysis (lead), methodology (supporting), writing – review and editing (equal). **Stefano Stagi:** conceptualization (lead), data curation (lead), formal analysis (lead), methodology (lead), supervision (lead), validation (lead), writing – review and editing (lead).

## Ethics Statement

The study was conducted in accordance with the Declaration of Helsinki and approved by the Ethics Committee of Anna Meyer Children's Hospital of Florence (protocol code 82/2021 approved on 23 March 2021).

## Conflicts of Interest

The authors declare no conflicts of interest.

## Data Availability

Data available on request.
